# Use of Vaginal Dinoprostone (PGE_2_) in Patients with Premature Rupture of Membranes (PROM) Undergoing Induction of Labor: A Comparative Study

**DOI:** 10.3390/jcm11082217

**Published:** 2022-04-15

**Authors:** Nuria López-Jiménez, Fiamma García-Sánchez, Rafael Hernández Pailos, Valentin Rodrigo-Álvaro, Ana Pascual-Pedreño, María Moreno-Cid, Antonio Hernández-Martínez, Milagros Molina-Alarcón

**Affiliations:** 1 Department of Obstetrics and Gynecology, La Mancha Centro Hospital, 13600 Alcazar de San Juan, Spain; nurialj92@gmail.com (N.L.-J.); fiammagss@gmail.com (F.G.-S.); rhdezalcalapailos@yahoo.es (R.H.P.); biovalen.r@gmail.com (V.R.-Á.); ginemancha@hotmail.com (A.P.-P.); mmorenocid@gmail.com (M.M.-C.); 2 Department of Nursing, Physiotherapy and Occupational Therapy, Faculty of Nursing, University of Castilla-La Mancha IDINE, Camilo José Cela, 14, 13071 Ciudad Real, Spain; 3 Department of Nursing, Physiotherapy and Occupational Therapy, Faculty of Nursing, University of Castilla-La Mancha IDINE, Av. de España, s/n, 02001 Albacete, Spain; milagros.molina@uclm.es

**Keywords:** premature rupture of membranes (PROM), induction of labor (IoL), PGE_2_, cesarean section, delivery time

## Abstract

Purpose: To evaluate the effect and safety of vaginal dinoprostone in pregnant women with PROM who undergo induction of labor (IoL). Materials and Methods: Prospective observational study conducted at La Mancha Centro hospital from 1 February 2019, to 30 August 2020. Obstetric and neonatal variables of 94 pregnant women with PROM who underwent IoL with vaginal dinoprostone were analyzed, and the results were compared with 330 patients without PROM who also underwent IoL. Bivariate and multivariate analyses were performed using binary and multiple linear regression. Results: A total of 424 women were included in this study. A greater response to cervical ripening (Bishop score > 6) with PGE_2_ was observed in the PROM group (odds ratio (OR) 2.73, 95% confidence interval (CI) 1.50–4.99, *p* = 0.001), as well as a shorter total duration of IoL (mean difference (MD) 2823.37 min (min), 95% CI 1257.30–4389.43, *p* < 0.001). Cesarean sections were performed in 28.7% (*n* = 27) of patients in the PROM group vs. 34.2% (*n* = 113) of patients in the non-PROM group, with no significant differences (OR 0.87%, 95% CI 0.47–1.60, *p* = 0.652). There were no significant differences in changes in the cardiotocographic record (CTG), postpartum hemorrhage (PPH), uterine rupture, or adverse neonatal outcomes between the two groups. Conclusions: The use of vaginal dinoprostone in pregnant women undergoing IoL with PROM is safe for the mother and the fetus, shortens the total delivery time, and does not increase the risk of cesarean section compared with pregnant women undergoing IoL without PROM.

## 1. Introduction

Premature rupture of membranes (PROM) is defined as the rupture of the fetal membranes before the onset of regular uterine contractions [1]. This condition can occur in term fetuses (≥37 weeks of gestation) and preterm fetuses (PPROM, <37 weeks of gestation) with an incidence of approximately 8% and 3%, respectively [2]. Its etiology is multifactorial, but the gestational age at which it occurs provides information regarding the underlying cause. Regarding full-term fetuses, it may be due to physiological weakening of the membranes combined with the shear forces created by uterine contractions with the onset of labor [1]. In the case of preterm fetuses, its association with intra-amniotic infection has been reported in 25–40% of pregnant women [3,4,5].

Induction of labor (IoL) is currently one of the most frequently performed procedures in obstetrics departments. In Europe, the induction rate varies between 6.8 and 33% [6]. The latest review carried out by Cochrane in 2017 was in favor of induction of labor (IoL) for PROM at term after demonstrating a reduction in the risk of maternal infectious and neonatal morbidity compared with expectant management [2]. However, it did not establish a recommendation for the safest induction method for this (oxytocin or prostaglandins); hence, its management remains controversial. Furthermore, several studies, including meta-analyses of randomized clinical trials, have not shown a statistically significant benefit in women with PROM for using any type of prostaglandin versus oxytocin [7,8,9,10]. By contrast, some studies have documented the efficacy and safety of the use of vaginal dinoprostone prior to the use of oxytocin for labor induction, with an increase in the rate of vaginal delivery within 24 h compared with labor induction with IV oxytocin alone (78.5% vs. 63.3%; relative risk (RR) 1.23; 95% CI, 1.09–1.39; *p* = 0.01) [11].

One of the most feared effects of the use of prostaglandins in patients with ruptured membranes is uterine hyperstimulation (6.2%) and the possible associated disorders regarding the fetal heart rate (FHR) (6.9%) [12]. Although societies such as the SEGO (Spanish Society of Obstetrics and Gynecology) [13] and the ACOG (American College of Obstetricians and Gynecologists) [1] do not contraindicate the use of dinoprostone to initiate labor induction in patients with unfavorable Bishop scores, it is remarkable to note that the Propess^®^ product data sheet recommends not using this product in pregnant women with PROM because of the limited experience of its use in this population and the possibility that its release is higher and more variable than in patients without PROM [12,14], possibly entailing more risks than in patients with intact membranes.

Therefore, to provide more evidence regarding the effect and safety for the mother and the fetus of vaginal dinoprostone in the induction of labor in patients with PROM, we analyzed and compared the perinatal results in pregnant women with PROM undergoing IoL in the first 24 h vs. pregnant women undergoing IoL without PROM.

## 2. Materials and Methods

### 2.1. Patients and Methods

A prospective observational study was carried out from 1 February 2019 to 20 August 2020 at La Mancha Centro hospital in Alcázar de San Juan (Ciudad Real), Spain. This study was approved by the hospital’s clinical research ethics committee (CEIC) with protocol number 102-C. All patients who participated in the study did so voluntarily and anonymously after agreeing to participate and signing an informed consent form.

The study population included a total of 424 singleton pregnancies undergoing IoL with vaginal dinoprostone (PGE_2_). A comparative study was conducted between patients undergoing IoL with PROM (*n* = 94) and patients with IoL for other reasons (*n* = 330) without PROM.

### 2.2. Inclusion and Exclusion Criteria

The inclusion criteria were singleton pregnancies with cephalic presentations, ≥34 weeks of gestation, a baseline Bishop score ≤6 points, and the need for cervical ripening prior to the induction of labor with vaginal dinoprostone (PGE_2_). There were no restrictions regarding parity or history of previous cesarean section. Patients with labor induction without PGE_2_, multiple gestations, noncephalic presentations, and stillbirths, along with patients who did not consent to participate in the study, were excluded.

### 2.3. Information Sources

For data collection, a specific computerized database was created. The information was obtained from personal clinical interviews and the data recorded in the partograms and medical records of the patients. Cervical characteristics prior to induction (Bishop score) and the cervical length measured by transvaginal ultrasound were recorded by the gynecologist responsible for the delivery room that day. The guidelines of the International Society of Ultrasound in Obstetrics and Gynecology (ISUOG) [15] and the Fetal Medicine Foundation (FMF) [16] were used for the measurement of cervical length. The main independent variable analyzed was the existence of the premature rupture of membranes (PROM), while the dependent variables were the obstetric and neonatal results obtained after IoL. Table 1 shows the sociodemographic and obstetric characteristics of the pregnant women studied according to the presence or absence of PROM.

### 2.4. Induction of Labour Protocol at the Study Center

Pregnant women who presented with an unfavorable cervix at the beginning of the IoL (Bishop score of ≤6) underwent a prior cervical ripening process with the administration of vaginal prostaglandins (PGE_2_) using the Propess^®^ slow-release system (Ferring Pharmaceuticals, Saint-Prex, Switzerland). This system contains 10 mg of PGE_2_ released at a rate of 0.3 mg/h over 24 h.

Fetal indications for IoL included the following: non-reassuring fetal heart rate (NRFHR), oligohydramnios, polyhydramnios, fetal growth restriction (FGR), small for gestational age, and macrosomia. Maternal indications for IoL included the following: maternal diseases, such as gestational or pregestational diabetes, cholestasis, chronic hypertension or hypertensive diseases of pregnancy, and poor obstetric history.

The diagnosis of patients with PROM was made by vaginal examination, after observing the leakage of amniotic fluid through the cervical orifice and its accumulation in the posterior vaginal fornix, or by a positive test for the protein insulin-like growth factor-binding 1 (IGFBP-1) in the vaginal fluid. These patients underwent IoL starting from 34 weeks of gestation. We carried out prior cervical ripening with PGE_2_ if the patient had a Bishop score of ≤6 and the rupture of membranes occurred within the first 24 h. Antibiotic prophylaxis was only administered for GBS infection if the patient had a positive screening or if the screening status was unknown.

Once the vaginal device was placed, the pregnant woman underwent fetal heart rate (FHR) monitoring for 2 h. After insertion, if any alteration in the FHR pattern (according to the classification criteria for FHR tracings proposed by the National Institute of Child Health and Human Development, NICHD) [17] or uterine tachysystole (defined as the presence of more than five contractions every 10 min) was observed, the device was removed immediately. If no alteration occurred, FHR monitoring was performed at 12 and 24 h, and the device was removed when the woman reached favorable cervical ripening (Bishop score of >6), dilation of 3–4 cm with regular uterine contractions, or after 24 h regardless of the Bishop score. By contrast, in selected cases, women with a favorable Bishop score at the beginning of induction (>6) were directly stimulated with intravenous oxytocin perfusion and amniotomy.

Specifically, oxytocin was administered intravenously via an infusion pump at a dose of 2 milliunits/minute with a period between dose increments of 15 min until reaching regular uterine dynamics (3–4 contractions/10 min) or a maximum dose of 20 milliunits/minute (120 mL/h).

### 2.5. Statistical Analysis

All analyses were conducted using the program SPSS v24.0 (Chicago, IL, USA). A descriptive analysis was performed with mean and standard deviation (SD) for quantitative variables and absolute and relative frequencies for categorical variables. We carried out a bivariate analysis to determine the sociodemographic and clinical differences between the group of women with PROM and the group of women without PROM. Bivariate and multivariate analyses were then carried out to analyze the effect of PROM on the different perinatal outcomes analyzed. For this, binary logistic regression or multiple linear regression was used depending on whether the result variable was categorical or quantitative in nature. On the basis of this, odds ratios (OR)/adjusted odds ratios (AOR) or mean differences (MD)/adjusted mean differences (aMD) were estimated with their respective 95% confidence intervals.

## 3. Results

**Figure 1:**
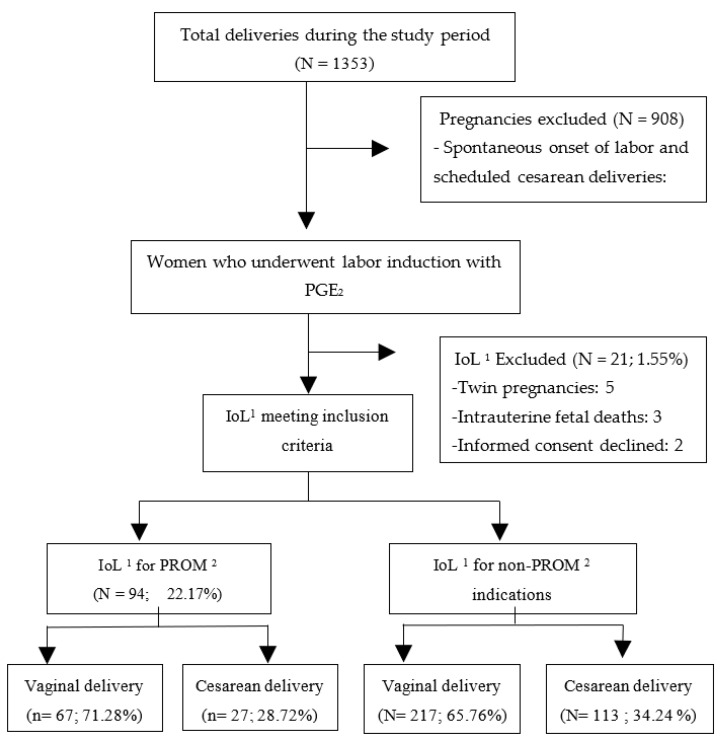
Flow chart of the selection process of the patients studied. ^1^ IoL: Induction of Labour. ^2^ PROM: Premature Rupture of Membranes.

A total of 1353 deliveries took place at La Mancha Centro hospital during the study period, of which 445 (32.89%) underwent IoL with PGE_2_. After applying the exclusion criteria, 424 pregnant women were recruited into the study. Of these, 94 (22.17%) underwent IoL for PROM, and 330 (77.83%) women underwent IoL for another indication without PROM. Figure 1 shows the flowchart of the patients included in the study.

### 3.1. Characteristics of the Women Undergoing IoL with PROM and without PROM

Both groups presented similar baseline variables. In the group of patients with PROM, the mean age was 32.29 years (SD = 4.83), and the pre-pregnancy BMI was 29.43 kg/m^2^ (SD = 4.70). In the non-PROM group, the mean age was 33.10 years (SD = 5.16), and the pre-pregnancy BMI was 30.20 kg/m^2^ (SD = 5.31). Regarding parity, a greater number of multiparous patients was observed in the PROM group (*n* = 73, 77.7%) vs. the non-PROM group (*n* = 207, 62.7%), although the result was non-significant (*p* = 0.07). Statistically significant differences were found regarding hypertension in pregnancy, which was more frequent in non-PROM patients (*n* = 38, 11.5% vs. *n* = 2, 2.1%; *p* = 0.043); intrauterine growth restriction, which was more frequent in patients with PROM (*n* = 20, 6.1% vs. *n* = 0.0%; *p* = 0.014); gestational age at birth (<37 weeks’ gestation), which was greater in the PROM group (*n* = 12, 12.8%) than in the non-PROM group (*n* = 3, 0.09%) (*p* < 0.001); cervical length at the beginning of the IoL, which was lower in the PROM group (21.27 mm, SD = 8.10) than in the non-PROM group (28.04 mm, SD = 9.11) (*p* < 0.001); and the prepartum Amniotic Fluid Index (AFI) (*p* = 0.025). No statistically significant differences were observed between both groups for the rest of the variables. Table 1 lists all the analyzed sociodemographic variables of the patients included in the study according to their PROM status.

### 3.2. Obstetric Outcomes According to PROM Status

A greater response to cervical ripening with PGE_2_ (Bishop score > 6) was observed in the group of patients with PROM vs. the non-PROM group (OR 2.73, 95% CI 1.50–4.99, *p* = 0.001) in the multivariate analysis. This difference was statistically significant for the nulliparous group, whereas it was not significant for the multiparous group. In addition, the time recorded with PGE_2_ and the total duration of IoL were significantly shorter in the PROM group: MD 1884.52 min (min) (95% CI 752.09–3016.96, *p* = 0.001) and MD 2823.37 min (95% CI 1257.30–4389.43, *p* < 0.001), respectively. A significantly higher risk of developing chorioamnionitis was observed in patients with PROM than in patients without PROM (OR 5.36, 95% CI 1.18–24.42, *p* = 0.030). Within the group of pregnant women with PROM, 71.28% (*n* = 67) had a vaginal delivery compared with 65.76% (*n* = 217) of the group without PROM, although this result was not significant (OR 0.87%, 95% CI 0.47–1.60, *p* = 0.652). No statistically significant differences were found in the bivariate or multivariate analyses between the two groups in terms of the presence of meconium, intrapartum fever, category II–III FHR pattern, postpartum hemorrhage (PPH), uterine rupture, or maternal admission to the ICU. All the obstetric variables studied are provided in detail in Table 2.

### 3.3. Neonatal Morbidity

In the group with PROM, APGAR test scores of <7 at 1 min were obtained in 3.2% (*n* = 3) of the neonates. No infants with APGAR scores of <7 at 5 min were recorded in this group. Regarding treatment, 12.8% (*n* = 12) required admission to the neonatal intensive care unit (NICU), and 3.2% (*n* = 3) required type III–IV neonatal resuscitation at birth. In the group of non-PROM pregnant women, APGAR scores of <7 at 1 min and 5 min were obtained in 2.7% (*n* = 9) and 0.6% (*n* = 2) of neonates, respectively. In terms of treatment, 10.3% (*n* = 34) required NICU admission, and 2.4% (*n* = 8) required type III–IV neonatal resuscitation at birth. No statistically significant differences were observed between the groups in the bivariate or multivariate analysis in any of the neonatal variables collected. Additionally, no cases of neonatal sepsis were recorded in either group, despite the intrapartum diagnosis of chorioamnionitis. Table 3 shows the neonatal results in the study population according to their PROM status in detail.

## 4. Discussion

The objective of this study was to evaluate the effect and safety of vaginal dinoprostone (PGE_2_) in pregnant women who underwent IoL with PROM. To do this, we prospectively compared the perinatal outcomes of 94 pregnant women needing to undergo IoL because of PROM vs. 330 patients requiring IoL without PROM. The results of this study report an increased response to cervical ripening with PGE_2_ in patients with PROM, as well as a shorter time needed with vaginal PGE_2_ and a shorter total duration of IoL compared with pregnant women undergoing labor induction without PROM. There were no significant differences between the two groups regarding the type of delivery, abnormalities in the CTG, uterine rupture, postpartum hemorrhage, low APGAR scores, or admission to the neonatal ICU. Although a higher risk of suspected intrapartum chorioamnionitis was observed in the PROM group (OR 5.36, 95% CI 1.18–24.42), no cases of neonatal sepsis were recorded in either group. 

Our results are consistent with those observed in the literature. Wang et al. [18] reported a shorter induction-to-delivery time in the PROM group vs. inductions without PROM with the use of vaginal dinoprostone (18.76 ± 13.03 h vs. 24.36 ± 17.75 h, *p* < 0.0001). They also reported a lower rate of cesarean sections (26.89 vs. 33.58%, *p* = 0.001) and a higher number of vaginal deliveries in 24 h (54.38 vs. 45.48%, *p* < 0.0001) and 48 h (71.90 vs. 63.16%, *p* <0.0001), without reporting any cases of maternal or fetal infection. Likewise, Kehl et al., reported similar results for the induction of labor with PROM with vaginal misoprostol and considered this to be a safe method [19]. In a review carried out by Matt Shirley in 2018 on the efficacy, safety, and tolerability of vaginal dinoprostone in IoL [20], fewer adverse events were reported with the use of PGE_2_ than with vaginal misoprostol preparations (11.4% vs. 4.0%, *p* < 0.001). A peer-reviewed meta-analysis published in 2015 found up to a threefold decrease in the risk of uterine hyperstimulation with vaginal dinoprostone use compared with vaginal misoprostol doses of ≥50 µg (OR 2.73, 95% CI 1.08–7.40) [21].

Regarding the finding of a higher risk of intrapartum chorioamnionitis in pregnant women with PROM, we consider that the time that the pregnant woman remains with ruptured membranes acts as an independent risk factor in the development of a possible intra-amniotic infection and should not be attributed to the effect of the vaginal dinoprostone itself [22,23]. Another important aspect to take into account is that the premature rupture of the membranes itself is an independent factor that stimulates the labor process and has been widely described [10]; hence, it can influence the time needed with Propess^®^, and the total IoL may end up being shorter compared with that in pregnant women without PROM.

Some studies, such as that by Lyrenäs et al. [14], have studied the rate of PGE_2_ release in patients undergoing IoL with both intact membranes and the premature rupture of membranes. In both groups, this release was observed to be safe and effective, without evidence of a “loading dose” (the release of an excessive amount of PGE_2_ over a short period of time). In patients with PROM, because of the increase in vaginal pH, a greater release of PGE_2_ by the device was observed. However, because of the changes in the ionization that the PGE_2_ molecule undergoes with an increase in pH, its vaginal absorption is lower, so the expected risk of uterine hyperstimulation is reduced.

This study has some limitations to consider. First, it is important to clarify that during the labor process, a suspicion of chorioamnionitis (also called intra-amniotic infection, IAI) [24] can only be established based on clinical criteria (fever of ≥39.0 °C (102.2 °F) or two measurements of ≥38 °C (102.02 °F) plus evidence of fetal tachycardia or a maternal white cell (WBC) count >15,000/mm^3^ in the absence of corticosteroids and ideally showing a left shift or purulent-appearing fluid coming from the cervical os visualized by speculum examination). For confirmation, the detection of Gram-positive bacteria in the amniotic fluid; a low level of amniotic glucose; or histopathological evidence of the infection of the placenta, fetal membranes, or the umbilical cord is needed [24]. A limitation of our study is the impossibility of obtaining a confirmatory diagnosis through a placental study after delivery in pregnant women with suspected IAI. However, we believe that there was no actual case, as no cases of neonatal infection/sepsis were observed in the population studied. On the other hand, we should note that a higher proportion of multiparous patients was observed for pregnant women with PROM, which may have influenced the shorter induction times. However, the authors believe that the risk of bias is low because a multivariate analysis was carried out to control for confounding bias. Finally, the sample size of our population is limited, which can make it difficult to obtain significant results for some rare adverse events, such as uterine rupture, which has a low reported frequency of occurrence [25].

Regarding strengths, it should be noted that this is a prospective study with well-defined and agreed-upon variables, as well as the use of a uniform induction protocol and well-detailed doses of oxytocin. In addition, taking into account the scant evidence on dinoprostone use for PROM, we believe that the results of this study could contribute to the performance of meta-analyses with greater statistical power to study rare adverse events, such as uterine rupture, thus enabling the establishment of strong recommendations on the most appropriate method of cervical ripening and induction in this group of patients.

## 5. Conclusions

Our results suggest that the use of vaginal dinoprostone (PGE_2_) in IoL in pregnant women with PROM is as safe as that in pregnant women without PROM, both for the mother and the fetus. A decrease in the total induction time was also observed, as well as a reduced time with dinoprostone in pregnant women with PROM, without significant differences observed in terms of the type of delivery compared with pregnant women without PROM.

## Figures and Tables

**Table 1:** Demographic characteristics of the study population according to PROM status.

Variable	PROM ^1^ (*n*= 94)	Non-PROM ^1^ (*n* = 330)	*p*-Value
**Maternal characteristics**			
Maternal age (years) *	32.29 (4.83)	33.10 (5.16)	0.174
Pregestational weight (kg) *	66.68 (12.95)	69.82 (14.84)	0.062
Antepartum weight (kg) *	78.47 (14.53)	80.82 (14.91)	0.178
Pregestational Body Mass Index (BMI) (kg/m^2^) *	29.43 (4.70)	30.20 (5.31)	0.203
**Obstetrical characteristics**			
**Previous cesarean delivery**			
No	85 (90.4%)	292 (88.5%)	0.597
Yes	9 (9.6%)	38 (11.5%)	
**Parity**			
Primiparity	21 (22.3%)	123 (37.3%)	0.007
Multiparity	73 (77.7%)	207 (62.7%)	
**Preexisting or gestational diabetes**			
No	91 (96.8%)	294 (89.1%)	0.154
Preexisting diabetes	0 (0%)	4 (1.2%)	
Gestational diabetes	3 (3.2%)	32 (9.7%)	
**Hypertensive state in pregnancy**			
No	92 (97.9%)	292 (88.5%)	0.043
Chronic hypertension	2 (2.1%)	6 (1.8%)	
Gestational hypertension	0 (0%)	18 (5.5%)	
Preeclampsia	0 (0%)	13 (3.9%)	
Preeclampsia with severe features	0 (0%)	1 (0.3%)	
**Fetal Growth Restriction (FGR)**			
No	94 (100%)	310 (93.9%)	0.014
Yes	0 (0%)	20 (6.1%)	
**Gestational age at birth (weeks)**			
<37 + 0 days	12 (12.8%)	3 (0.09%)	<0.001
≥37 + 0 days	82 (87.2%)	327 (99.1%)	
Cervical length prior to IoL ^2^ (CL) *, millimeters (mm)	21.27 (8.10)	28.04 (9.11)	<0.001
**Bishop score on admission**	2.13 (1.53)	2.81 (1.45)	<0.001
**Prepartum Amniotic Fluid Index (AFI)**			
Normal	78 (83%)	270 (81.8%)	0.025
Oligoamnios	15 (16%)	34 (10.3%)	
Hydramnios	1 (1.1%)	26 (7.9%)	

* Mean (Standard Deviation). ^1^ PROM: Premature Rupture of Membranes. ^2^ IoL: Induction of Labor.

**Table 2:** Obstetric outcomes in the study population according to PROM.

Variable	PROM ^1^ (*n* = 94)	Non-PROM ^1^ (*n* = 330)	Univariate Analysis		Multivariate Analysis **	
			OR/MD 95% CI	*p*-Value	OR/MD 95% CI	*p*-Value
**Bishop score >6 after PGE_2_**						
No	26 (27.7)	161 (48.8)	2.49 (1.51–4.11)	<0.001	2.73 (1.50–4.99)	0.001
Yes	68 (72.3)	169 (51.2)				
**Nulliparous Bishop score >6 after PGE_2_**						
No	19 (20.2)	116 (35,2)	3.62 (2.01–6.54)	<0.001		
Yes	54 (57.45)	91 (27.6)				
**Multiparous Bishop score >6 after PGE_2_**						
No	7 (7.45)	45 (13.6)	1.15 (0.43–3.07)	0.774		
Yes	14 (14.9)	78 (23.6)				
Time with PGE_2_ (min) *	418.51 (310.22)	792.42 (420.50)	373.92 (281.40–466.43)	<0.001	1884.52 (752.09–3016.96)	0.001
Total duration of IoL (min) *	903.03 (525.73)	1345.38(653.9)	442.35 (297.29–597.41)	<0.001	2823.37 (1257.30–4389.43)	<0.001
Duration of 2nd stage (min) *	104.50 (82.61)	90.56 (78.35)	−13.94 (−35.79–7.83)	0.209	−274.51 (−542.58–6.43)	0.45
**Type of delivery**						
Vaginal delivery	67 (71.3)	217 (65.8)	0.77 (0.47–1.28)	0.316	0.87 (0.47–1.60)	0.652
Cesarean delivery	27 (28.7)	113 (34.2)				
**Meconium**						
No	86 (91.5)	278 (84.2)	0.50 (0.23–1.09)	0.080	0.64 (0.26–1.57)	0.327
Yes	8 (8.5)	52 (15.8)				
**Reason for cesarean delivery**						
Arrest of labor	12 (12.8)	27 (8.2)	1.64 (0.80–3.38)	0.178	2.32 (0.95–5.69)	0.066
Failed induction	4 (4.3)	32 (9.7)	0.41 (0.14–1.20)	0.105	0.51 (0.15–1.74)	0.282
Cephalopelvic disproportion	5 (5.3)	11 (3.3)	1.63 (0.55–4.81)	0.377	1.42 (0.40–5.12)	0.588
Emergent	6 (6.4)	43 (13)	0.45 (0.19–1.10)	0.082	0533 (0.20–1.41)	0.206
**Intrapartum fever**						
No	88 (93.6)	313 (94.8)	0.75 (0.28–1.97)	0.560	0.60 (0.20–1.80)	0.362
Yes	6 (6.4)	16 (4.8)				
Missing data		1 (0.3)				
**Intrapartum chorioamnionitis**						
No	89 (94.7)	323 (97.9)	3.03 (0.91–10.17)	0.072	5.36 (1.18–24.42)	0.030
Yes	5 (5.3)	6 (1.8)				
Missing data		1 (0.3)				
**CTG ^2^: NICHD ^3^ 2**						
No	68 (72.3)	234 (70.9)	0.93 (0.55–1.55)	0.787	0.90 (0.51–1.57)	0.702
Yes	26 (27.7)	96 (29.1)				
**CTG ^2^: NICHD ^3^ 3**						
No	91 (96.8)	312 (94.5)	0.57 (0.17–1.98)	0.378	0.55 (0.15–2.06)	0.372
Yes	3 (3.2)	18 (5.5)				
**Postpartum hemorrhage ^4^**						
No	87 (92.6)	304 (92.1)	1.06 (0.44–2.56)	0.891	0.91 (0.33–2.51)	0.852
Yes	7 (7.4)	23 (7.0)				
Missing data		3 (0.9)				
**Uterine rupture ^5^**						
No	93 (98.9)	327 (99.1)	3.52 (0.29–56.75)	0.376	NC	0.994
Yes	1 (0.3)	1 (0.3)				
Missing data		2 (0.6)				
**Blood loss >3.5 (l)**						
No	90 (95.7)	308 (93.3)	0.57 (0.16–1.98)	0.377	0.84 (0.21–3.45)	0.814
Yes	3 (3.2)	18 (5.5)				
Missing data	1 (1.1)	4 (1.2)				
**ICU ^6^ admission**						
No	93 (98.9)	326 (98.8)	3.50 (0.22–56.58)	0.377	0.67 (0–54.31)	0.858
Yes	1 (1.1)	1 (0.3)				
Missing data		3 (0.9)				
**Need for transfusion**						
No	90 (95.7)	318 (96.4)	1.32 (0.34–5.10)	0.682	1.57 (0.30–8.13)	0.592
Yes	3 (3.2)	8 (2.4)				
Missing data	1 (1.1)	4 (1.2)				

**OR**: odds ratio; **MD**: mean difference; **CI**: confidence interval. ^1^ PROM: Premature rupture of membranes. **^2^ CTG**: cardiotocography. **^3^ NICHD**: CTG classification based on the system proposed by the National Institute of Child Health and Human Development. **^4^ Postpartum hemorrhage**: defined as more bleeding than expected with signs and symptoms of hypovolemia, for which the gynecologist had to initiate uterotonic drugs. **^5^ Uterine rupture**: complete rupture of all uterine layers, including the serosal layer. **^6^ ICU**: Intensive Care Unit. **NC**: Not calculated. * Mean (Standard Deviation). ** Multivariate analysis adjusted for previous cesarean delivery, maternal age, pregestational BMI, Bishop score at admission, neonatal weight, regional analgesia, oxytocin stimulation, parity, hypertensive state in pregnancy, preexisting or gestational diabetes, and gestational age.

**Table 3:** Neonatal outcomes in the study population according to PROM.

Variable	PROM ^5^ (*n* = 94)	Non-PROM ^5^ (*n* = 330)	Univariate Analysis		Multivariate Analysis **	
			OR 95% CI	*p*-Value	OR 95% CI	*p*-Value
**APGAR ^1^ score <7 at 1 min**						
No	91 (96.8%)	321 (97.3%)	1.18 (0.31–4.43)	0.811	1.70 (0.37–7.83)	0.494
Yes	3 (3.2%)	9 (2.7%)				
**APGAR score <7 at 5 min**						
No	94 (100%)	328 (99.4%)	NC	0.997	2.54 (NC)	1
Yes	0 (0%)	2 (0.6%)				
**NICU ^2^ admission**						
No	82 (87.2%)	296 (89.7%)	0.78 (0.39–1.58)	0.499	1.24 (0.47–3.28)	0.661
Yes	12 (12.8%)	34 (10.3%)				
**REA ^3^ III–IV**						
No	91 (96.8%)	322 (97.6%)	1.33 (0.34–5.10)	0.681	3.77 (0.68–21.33)	0.133
Yes	3 (3.2%)	8 (2.4%)				
**U. artery ^4^ pH <7.20 at birth**						
No	86 (91.5)	282 (85.5)	0.50 (1.19–1.31)	0.158	0.87 (0.30–2.54)	0.795
Yes	5 (5.3)	33 (10.0)				
Missing data	3 (1.2)	15 (4.5)				
**U. artery ^4^ pH < 7.10 at birth**						
No	90 (95.8)	313 (94.85)	1.74 (0.16–19.40)	0.653	NC	0.957
Yes	1 (0.3)	2 (0.6)				
Missing data	3 (1.2)	15 (4.55)				

**OR**: odds ratio; **CI**: confidence interval. ^1^**APGAR**: Scoring system for the newborn (Appearance, Pulse, Grimace, Activity, Respiration). **NICU ^2^**: Neonatal intensive care unit. **^3^ REA**: Degree of neonatal resuscitation required at birth. **^4^ U. artery**: Umbilical artery. **^5^ PROM**: Premature rupture of membranes. ** Multivariate analysis adjusted for neonatal weight, NICHD classification, meconium, intrapartum fever, prematurity, previous cesarean delivery, general anesthesia, Fetal Growth Restriction (FGR), hypertensive state in pregnancy, preexisting or gestational diabetes, oxytocin stimulation, and gestational age.

## Data Availability

The datasets generated and/or analyzed during the current study are available from the corresponding author on reasonable request.
